# Life-Threatening Cardiac Compression Secondary to a Massive Hiatus Hernia With Gastric Volvulus: A Case Report

**DOI:** 10.7759/cureus.90402

**Published:** 2025-08-18

**Authors:** Adi Ahmed, Samraiz Nafees, Ali Javeed, Layal Ahmed Ali

**Affiliations:** 1 Emergency Medicine, Scarborough General Hospital, Scarborough, GBR; 2 Internal Medicine, Scarborough General Hospital, Scarborough, GBR; 3 Medicine and Surgery, Norwich Medical School, Norwich, GBR

**Keywords:** cardiac tamponade, cardiology, cardiothoracics, cardiovascular, emergency, esophagogastroduodenoscopy (ogd), hemodynamic compromise, pericardial effusion, surgical gastro, volvulus

## Abstract

Large hiatal hernias are typically managed conservatively; however, in rare instances, they can lead to life-threatening mechanical cardiovascular complications through direct extrinsic compression of the cardiac chambers rather than pericardial fluid accumulation, producing “functional” tamponade physiology. An 88-year-old man presented to the emergency department with sudden worsening of a three-day history of chest and upper abdominal pain radiating to the back. On arrival, he was vomiting, dyspneic, pale, and hypotensive (blood pressure: 96/52 mmHg). A CT angiogram revealed a massive hiatal hernia containing the stomach and transverse colon, complicated by gastric volvulus and anterior cardiac compression consistent with tamponade physiology. Nasogastric decompression was unsuccessful due to distorted anatomy. The patient subsequently underwent an urgent esophagogastroduodenoscopy (OGD) within one hour of CT acquisition, which aspirated 1.1 L of thick gastric contents, leading to the resolution of the cardiac compression. OGD was chosen over immediate surgical intervention due to the patient’s frailty and comorbidities, with the aim of rapid decompression and minimizing the perioperative risk. He made a full recovery and was discharged shortly thereafter, remaining symptom-free at the three-month follow-up. This case highlights a rare but critical complication of massive hiatal hernia causing functional cardiac tamponade. Prompt recognition through CT imaging and timely endoscopic decompression can be life-saving.

## Introduction

Hiatal hernias are a relatively common gastrointestinal abnormality, particularly in elderly populations, with an estimated prevalence of up to 60% in individuals over the age of 60 years [1]. Hiatal hernias are classified into the following four main types: type I (sliding), where the gastroesophageal junction and part of the stomach herniate into the thorax; type II (true para-esophageal), where the gastric fundus herniates alongside a normally positioned gastresophageal junction; type III, which combines elements of both; and type IV, where other abdominal viscera (such as colon or spleen) herniate through the hiatus [1]. Most hiatal hernias are asymptomatic or cause minor symptoms such as reflux or dysphagia and are managed conservatively. However, in rare instances, large hiatal hernias can lead to life-threatening complications, including gastric volvulus, obstruction, strangulation, or, in exceedingly rare cases, cardiac compression [2].

Massive intrathoracic herniation of abdominal viscera, particularly the stomach and colon, can distort mediastinal anatomy, exerting extrinsic pressure on adjacent structures, such as the heart, great vessels, and lungs. This can result in a phenomenon sometimes referred to as “functional cardiac tamponade,” defined as hemodynamic compromise due to impaired diastolic filling from external compression of the heart, most often the right atrium and ventricle, without the presence of pericardial effusion [3,4]. Right-sided chambers are more susceptible because of their thinner walls and lower filling pressures, making them more prone to collapse under extrinsic pressure.

Several published reports have described cardiac compromise resulting from gastric distension in the setting of hiatal hernias, particularly in elderly patients with comorbidities [5-7]. However, these cases remain exceptionally rare, and diagnosis can be delayed due to overlapping symptoms with cardiac ischemia, pulmonary embolism, or acute abdomen.

In this report, we present the case of an 88-year-old man who developed hemodynamic instability due to cardiac compression by a massive hiatal hernia complicated by gastric volvulus. This case highlights the importance of early cross-sectional imaging, the potential for CT to identify extracardiac causes of tamponade physiology, and the need for multidisciplinary, time-sensitive management involving emergency physicians, radiologists, and surgeons. Our aim is to contribute to the growing but limited literature on this rare complication and emphasize the diagnostic and therapeutic considerations.

## Case presentation

An 88-year-old male with a medical history significant for a large known hiatal hernia, hypertension, asthma, and gastroesophageal reflux disease presented to the emergency department with a three-day history of progressively worsening central chest and upper abdominal pain radiating to his back. The patient also reported multiple episodes of non-bilious vomiting and increasing shortness of breath. His wife noted that he had appeared increasingly pale and lethargic over the preceding week.

On arrival, the patient was visibly frail and pale, exhibiting signs of respiratory distress with increased work of breathing. Vital signs demonstrated a blood pressure of 96/52 mmHg, heart rate of 110 beats per minute, respiratory rate of 24 breaths per minute, oxygen saturation of 91% on room air, and a temperature of 35.5°C. Physical examination revealed reduced air entry bilaterally without wheezing or crackles, and heart sounds were normal with no audible murmurs or pericardial rubs. The abdomen, however, appeared distended and was generally tender. The patient was in evident discomfort on palpation of all quadrants. No signs of peritonism were noted.

**Figure 1:**
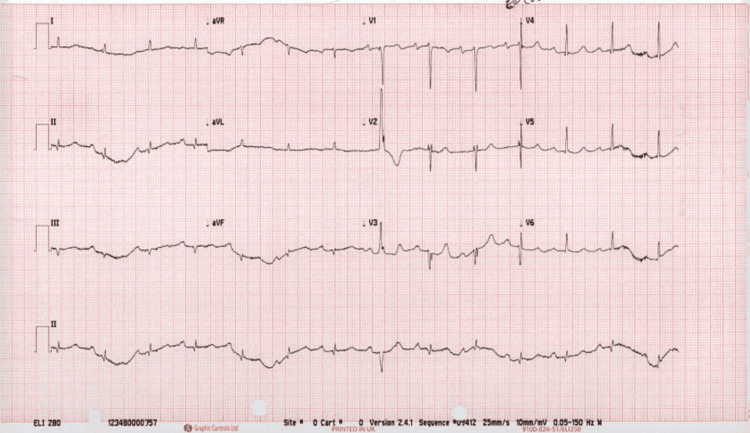
12-lead electrocardiogram (ECG) on presentation. The ECG demonstrates a normal sinus rhythm with a first-degree atrioventricular (AV) block (PR interval >200 ms). No acute ischemic changes or ST-segment deviations are observed. These findings, while non-specific, were interpreted in the context of the patient’s symptoms and hemodynamic compromise to support further urgent imaging.

Initial investigations included a 12-lead electrocardiogram (Figure 1), which revealed a sinus tachycardia with first-degree atrioventricular block but no ischemic changes. Blood tests revealed a mildly elevated lactate of 2.3 mmol/L and a troponin level of 14 ng/L that was non-dynamic on repeat testing, suggesting no acute myocardial infarction. Inflammatory markers, including white blood cell and neutrophil counts, were raised. C-reactive protein was mildly elevated at 20 mg/L (Table 1).

**Table 1:** Blood results. Table showing the full blood results for the patient. A repeat troponin came back as 14 ng/L.

Test name	Result	Units	Reference range
Hematology			
Hemoglobin	140	g/L	130–180
White cell count	17.5	×10⁹/L	4.0–11.0
Platelet count	339	×10⁹/L	150–450
Red cell count	4.70	×10¹²/L	4.30–5.80
Hematocrit	0.434	L/L	0.390–0.500
Mean cell volume	92	fL	80–100
Mean cell hemoglobin	29.8	pg	27.0–32.0
Neutrophils	14.70	×10⁹/L	2.00–8.00
Lymphocytes	1.47	×10⁹/L	0.50–4.00
Monocytes	1.05	×10⁹/L	0.20–1.20
Eosinophils	0.15	×10⁹/L	0.01–0.50
Basophils	0.05	×10⁹/L	0.00–0.10
Immature granulocytes	0.10	×10⁹/L	0.00–0.20
Coagulation			
Activated partial thromboplastin time	21.1	seconds	20.0–27.0
Prothrombin time	11.7	seconds	10.1–11.6
Fibrinogen	1.9	g/L	1.9–4.0
Biochemistry			
Bicarbonate	26	mmol/L	22–29
Urea	5.0	mmol/L	2.5–7.8
Sodium	140	mmol/L	133–146
Creatinine	74	μmol/L	59–104
Potassium	5.1	mmol/L	3.5–5.3
Troponin T	14	ng/L	Not provided
Estimated glomerular filtration rate (CKD-EPI)	70	mL/minute	>90 (normal)
Inflammation marker			
C-reactive protein	20	mg/L	<5 (normal)

**Figure 2:**
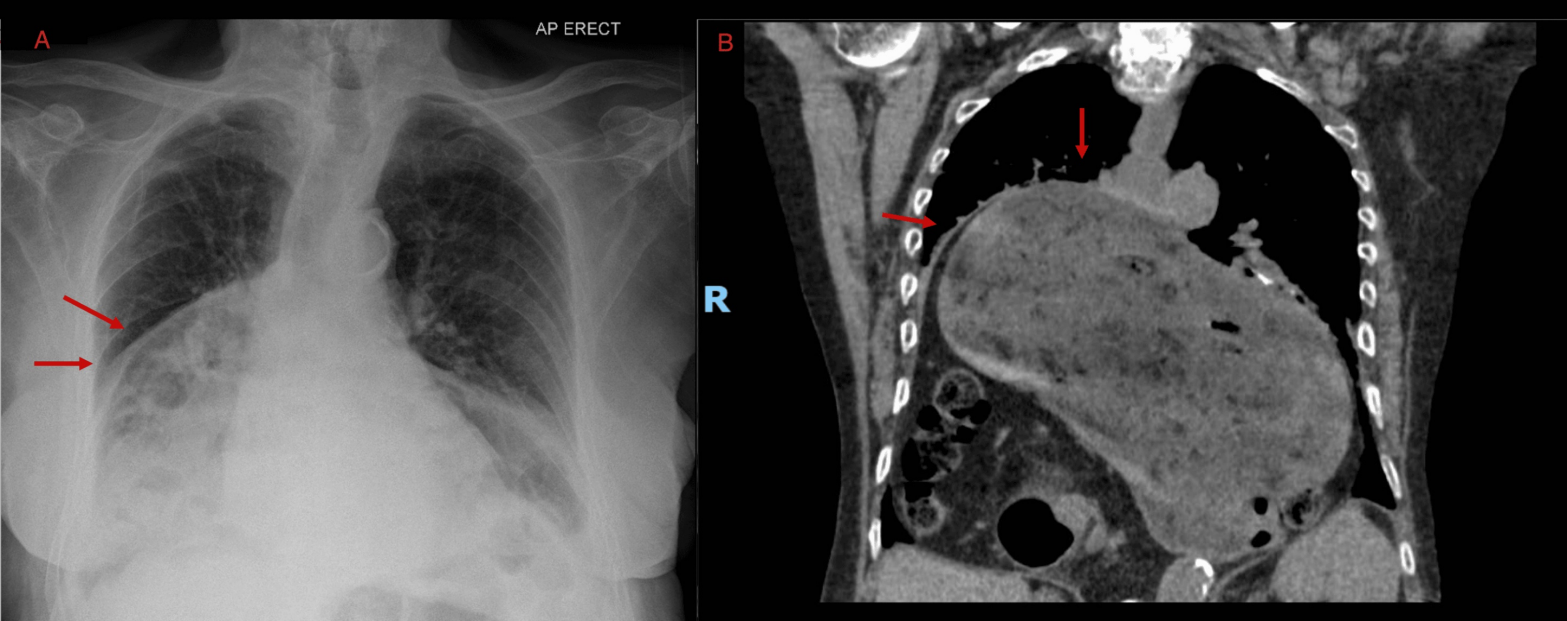
X-ray (A) and CT (B) scan of the chest. (A) Upright chest radiograph demonstrating a large retrocardiac air–fluid level, consistent with a massive hiatal hernia. The cardiac silhouette appears distorted and mildly displaced. (B) Coronal section from CT angiogram of the thorax showing a large hiatal hernia containing the stomach and transverse colon. The herniated viscera are seen exerting significant anterior compression on the heart, displacing it within the thoracic cavity. This mechanical effect mimicked tamponade physiology.

**Figure 3:**
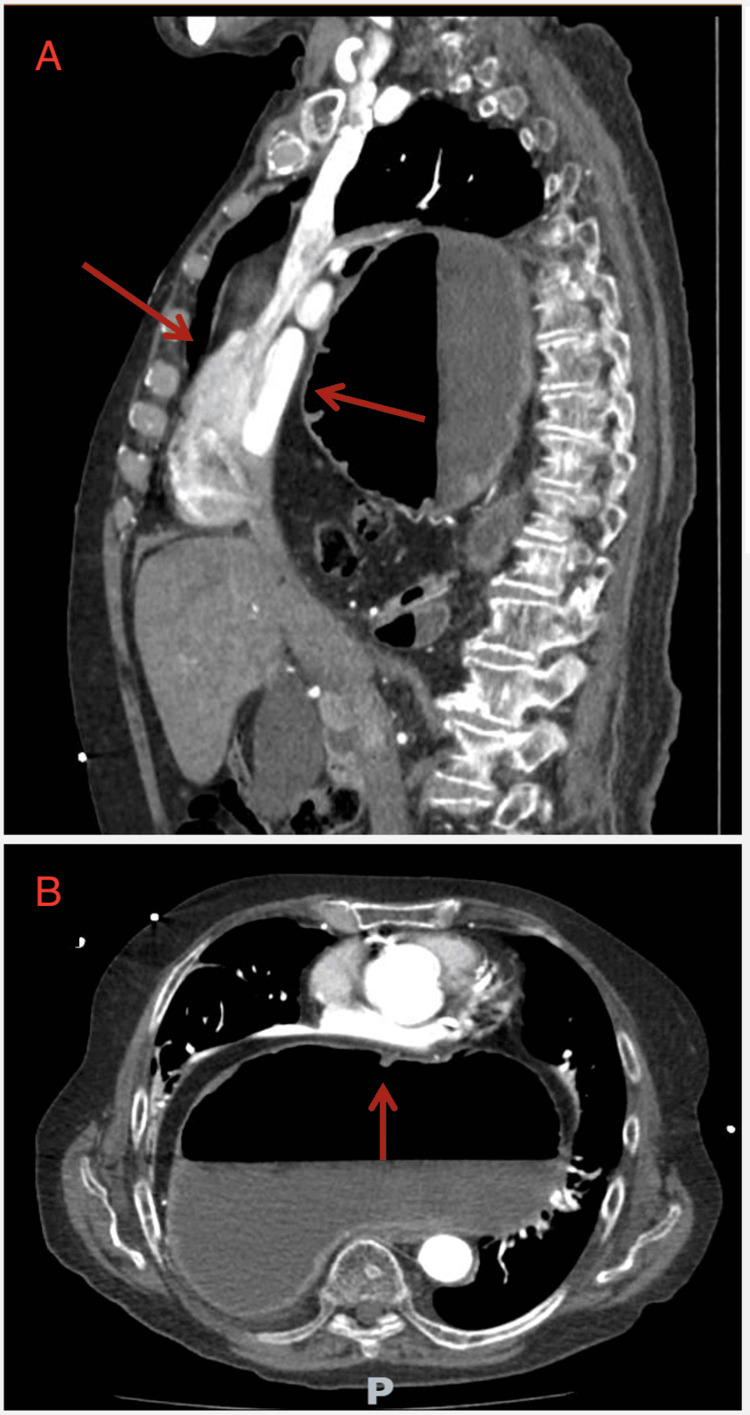
Sagittal (A) and Axial (B) view CT thorax. Pan-coronary atheromatous disease and compression of the heart (tamponade) due to massive overdistension of the hiatus hernia can be seen. Critical cardiac compromise was identified. Coronary artery patency was not evaluated on this CT study.

Given the clinical presentation and concern for acute aortic syndrome or pulmonary embolism, an urgent CT angiogram of the thorax, abdomen, and pelvis was performed. Imaging revealed a massive hiatal hernia containing both the stomach and transverse colon herniated into the mediastinum. The herniated contents were causing significant anterior displacement and compression of the heart against the sternum, producing a functional tamponade physiology by limiting cardiac preload and diastolic filling (Figures 2, 3). The CT also revealed twisting of the gastric outlet consistent with gastric volvulus. There was no evidence of aortic dissection, pulmonary embolism, or pericardial effusion. No echocardiography was performed, given the urgent need for intervention.

**Figure 4:**
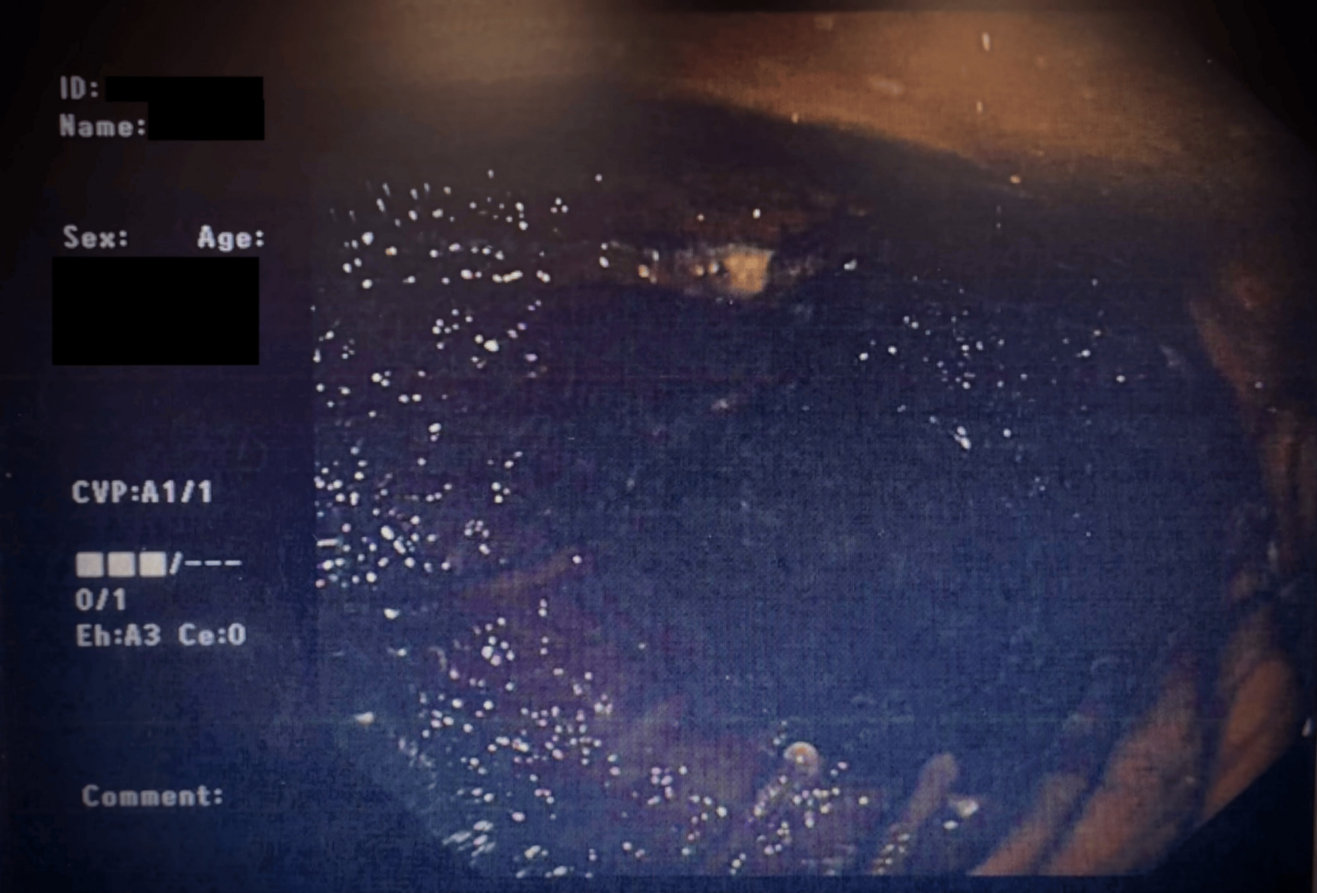
Endoscopic image following gastroscopy. Endoscopic image showing old digested food in the stomach which was subsequently aspirated. The gastric wall was noted to be pale, indicating ischemia.

Multiple attempts at nasogastric tube insertion for decompression failed due to the distorted anatomy and angulation of the esophagus. Given the hemodynamic instability and risk of ongoing cardiac compromise, the patient was taken urgently to the endoscopy suite for esphagogastroduodenoscopy (OGD) under general anesthesia within one hour of CT angiogram completion. Endoscopic findings included a short esophagus with a large, markedly distended stomach containing thick, retained secretions and food contents (Figure 4). The pale gastric wall raised concern for early ischemia; however, there was no evidence of necrosis or perforation, and a surgical review concurred that urgent decompression was the safest initial step. Approximately 1,100 mL of thick gastric fluid was aspirated during the procedure, leading to rapid decompression of the stomach and immediate improvement in hemodynamics.

Post-procedure, no complications from general anesthesia were observed despite the patient’s frailty. The patient’s blood pressure stabilized, respiratory distress resolved, and his symptoms of chest pain and vomiting abated. He was managed with intravenous fluids, antiemetics, and proton pump inhibitors. Serial cardiac monitoring and imaging confirmed resolution of cardiac compression, and no further ischemic or hemodynamic complications occurred.

The patient was discharged after a seven-day hospital stay with outpatient follow-up arranged with gastroenterology and surgical teams for evaluation of definitive management options for his hiatal hernia. At the three-month follow-up, he remained asymptomatic with no recurrent episodes of chest pain or dyspnea. Surgical repair was discussed but deferred due to high perioperative risk. In the end, conservative observation was chosen with instructions for urgent reassessment if symptoms recurred.

## Discussion

Hiatal hernias are a common clinical finding, especially in elderly populations, and are frequently managed conservatively unless complicated by symptoms such as reflux or obstruction. However, large or giant hiatal hernias may lead to rare but potentially life-threatening complications, including gastric volvulus, strangulation, and, as in this case, mechanical cardiovascular compromise due to extrinsic cardiac compression.

The phenomenon of cardiac tamponade typically involves pericardial fluid accumulation, restricting cardiac filling, and causing hemodynamic compromise. Functional tamponade physiology from external compression by a giant hiatal hernia is an uncommon and under-recognized entity. The mechanical pressure exerted by herniated abdominal contents can impair diastolic filling by compressing the cardiac chambers, particularly the right atrium and ventricle, leading to hypotension and reduced cardiac output, mimicking classic tamponade physiology without pericardial effusion. Right-sided chambers are especially vulnerable to external compression due to their thinner walls and lower filling pressures. Similar cases have been reported, emphasizing the importance of recognizing this rare presentation to avoid delays in treatment [6,7].

Estimates of the incidence of functional cardiac tamponade due to hiatal hernia are not well defined, but case series and reviews suggest it remains exceptionally rare, perhaps fewer than 1% of giant hiatal hernias complicated by volvulus result in cardiac compression.

Gastric volvulus associated with large hiatal hernias adds complexity and urgency to management. Volvulus occurs when the stomach twists on its axis, potentially causing obstruction, ischemia, and perforation if not promptly decompressed [7]. In our patient, the distorted anatomy rendered nasogastric decompression unsuccessful, necessitating urgent endoscopic intervention. Endoscopic decompression under general anesthesia allowed direct visualization and aspiration of significant retained gastric contents, effectively relieving the cardiac compression and restoring hemodynamic stability. The decision to proceed with OGD rather than immediate surgical repair was guided by the patient’s frailty, comorbidities, and the goal of rapid decompression with minimal perioperative risk.

The utility of CT imaging in diagnosing these complex presentations is well documented. CT angiography not only excludes life-threatening differentials such as aortic dissection and pulmonary embolism but also accurately delineates the extent of herniation, gastric volvulus, and cardiac compression [8]. Early imaging is crucial in elderly patients presenting with atypical chest pain and gastrointestinal symptoms, especially when known hiatal hernias exist.

Management strategies must be individualized, balancing surgical risks against the benefits of definitive repair. In frail elderly patients, non-operative management with endoscopic decompression and close monitoring may be preferable initially. However, surgical repair of the hiatal hernia can be considered for definitive treatment, especially in recurrent or complicated cases [9,10]. Delaying surgery carries risks of volvulus recurrence or re-compression, but may be justified in high-risk patients where conservative management is safer. Long-term recurrence risk remains uncertain, highlighting the need for close follow-up.

This case highlights the importance of a multidisciplinary approach, involving emergency physicians, gastroenterologists, radiologists, and surgeons. Prompt recognition via imaging and intervention can be lifesaving in the rare event of cardiac tamponade physiology secondary to hiatal hernia and gastric volvulus.

Learning points

Large hiatal hernias can cause rare but severe complications, including mechanical cardiac tamponade due to extrinsic cardiac compression. Additionally, gastric volvulus associated with hiatal hernias requires urgent recognition and decompression to prevent ischemia and cardiac compromise; nasogastric decompression may fail due to distorted anatomy, in which case urgent endoscopic decompression is a critical therapeutic option. Furthermore, functional cardiac tamponade without pericardial effusion should be considered in patients with hemodynamic instability and large mediastinal hernias compressing the heart. Early CT imaging is essential for accurate diagnosis, exclusion of other life-threatening conditions, and guiding management. Finally, a multidisciplinary approach is vital for optimal outcomes in complex cases involving overlapping cardiac and gastrointestinal pathology.

## Conclusions

This case illustrates a rare but potentially fatal complication of a massive hiatal hernia causing functional cardiac tamponade through extrinsic compression of the heart, compounded by gastric volvulus. Early recognition through prompt imaging and timely endoscopic decompression was lifesaving in this elderly patient. In frail patients, endoscopic intervention may offer immediate decompression with lower perioperative risk, while deferring surgery for later consideration. Clinicians should maintain a high index of suspicion for mechanical cardiac compromise in patients with large hiatal hernias presenting with chest pain and hemodynamic instability. A multidisciplinary approach is essential to optimize diagnosis and patient outcomes in such complex clinical scenarios.
